# Fecal occult blood test screening uptake among immigrants from Muslim majority countries: A retrospective cohort study in Ontario, Canada

**DOI:** 10.1002/cam4.2541

**Published:** 2019-09-30

**Authors:** Mandana Vahabi, Aisha Lofters, Josephine Pui‐Hing Wong, Lisa Ellison, Erin Graves, Cynthia Damba, Richard H. Glazier

**Affiliations:** ^1^ Faculty of Community Services Daphne Cockwell School of Nursing Ryerson University Toronto ON Canada; ^2^ Graduate Program in Immigration and Settlement Studies Ryerson University Toronto ON Canada; ^3^ Ryerson Centre for Global Health and Health Equity Toronto ON Canada; ^4^ Institute for Clinical Evaluative Sciences Toronto ON Canada; ^5^ Centre for Urban Health Solutions Li Ka Shing Knowledge Institute St. Michael's Hospital Toronto ON Canada; ^6^ Department of Family and Community Medicine University of Toronto Toronto ON Canada; ^7^ Department of Family and Community Medicine St. Michael Hospital Toronto ON Canada; ^8^ Dalla Lana School of Public Health University of Toronto Toronto ON Canada; ^9^ Toronto Central Local Health Integration Toronto ON Canada

**Keywords:** access to primary care, Colorectal cancer, Muslim immigrants, fecal occult blood test, region of origin, Social determinants of health

## Abstract

**Background:**

Colorectal cancer (CRC) is the second and third highest cause of cancer deaths among Canadian men and women, respectively. Population‐based screening through fecal occult blood testing (FOBT) has been proven to be effective in reducing CRC morbidity and mortality. Although participation in Ontario's organized CRC screening program has been increasing steadily since 2008, its uptake remains low among recent immigrant populations despite the known benefits of screening. To promote participation in CRC screening, it is imperative to understand both individual and system level barriers and enablers. Although a number of immigrant and nonimmigrant factors have been associated with low participation, there is a dearth of knowledge related to the religious affiliation in CRC screening uptake. Our study is among the first to examine this issue in Ontario, one of the most ethnically diverse Canadian provinces and preferred settlement destinations for immigrants.

**Methods:**

We conducted a population‐based retrospective cohort study using linked health care administrative databases. Our cohort included Ontario residents, age 50‐74 who were eligible for FOBT from 1 April 2013 to 31 March 2015.

**Results:**

We found that immigrants from the Middle East and North Africa and Eastern Europe and Central Asia had the lowest rates of screening. Furthermore, being born in a Muslim‐majority country was associated with lower FOBT screening even after controlling for other confounders including world region and income (ie, overall adjusted relative risk (ARR) of screening 0.92 [95% CI 0.90‐0.93]). Moreover, being enrolled in a primary care model, having a female primary care provider and having an internationally trained physician were associated with increased screening among immigrants from Muslim‐majority countries.

**Conclusions:**

These findings can inform future efforts to improve screening uptake like: enhancing access to primary care providers and enrollment in primary care models, targeted FOBT education for male providers and providers not in a primary care model, development of culturally sensitive and appropriate educational materials, and use of interactive approaches for communication of cancer screening information.

## Graphical abstract

The FOBT screening uptake was low among immigrants from Muslim‐majority country. Immigrants from the Middle East and North Africa and Eastern Europe and Central Asia countries were at high risk of under‐screening. FOBT uptake was lower for people with no primary care provider and high income. Having a female primary care provider and having an internationally trained physician were associated with higher FOBT use among immigrants from Muslim majority countries.

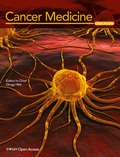

## 1 BACKGROUND

Colorectal cancer (CRC) is one of the most common types of cancers and a leading cause of cancer deaths in Canada.^1^ It is accountable for approximately 13% of new cancer cases and 12% of cancer deaths.^2^ Population‐based CRC screening is highly effective in early detection of the disease and reducing mortality and health care costs that are associated with more complex and invasive treatments.^3^, ^4^

Fecal occult blood testing (FOBT) is an integral part of screening programs in Canada. In Ontario, ColonCancerCheck (CCC), the province's organized population‐based screening program, offers FOBT every 2 years to asymptomatic adults aged 50‐74 years without a family history of CRC (ie, at average risk of developing colorectal cancer), followed by colonoscopy within 8 weeks in the event that the FOBT result is abnormal.^5^ Since June 2019, the program has switched to fecal immunochemical testing as the recommended screening test. Although colonoscopy every 10 years is fully covered under universal health care in Ontario, it is not recommended for average‐risk individuals as a screening modality by either Cancer Care Ontario (CCO) or the Canadian Taskforce on Preventive Health Care. The FOBT is a self‐sampling of feces from three separate bowel movements onto small piece of paper which is then placed into a prepaid envelope and mailed to the lab for testing. CCC actively targets eligible adult Ontarians by sending them invitation letters to discuss CRC screening with their primary care providers and dispensing FOBT kits to physicians for distribution to their patients. FOBT kits can also be accessed through nurse practitioners or Telehealth Ontario. Since the launch of the CCC program in 2008, the Ontarian participation in CRC screening has been increasing steadily, reaching 61% in 2015,^6^ however its uptake remains low among the province's immigrant population.^7^

Immigrants face more challenges than the general population in accessing screening services. Their health may take a back burner to settlement issues such as securing a job, finding affordable shelter, learning a new language, and creating social networks. Ontario is the most diverse and highly populated province in the country, and the preferred settlement destination for a considerable proportion of immigrants and refugees in Canada. More than half of Canadian immigrants and refugees reside in Ontario.^8^ The main source regions of immigrants and refugees in Canada have substantially changed over the past few decades mainly due to economic, social, and political circumstances in these source regions. In 2016, approximately 48% of the immigrants were born in Asia (including the Middle East)—an increase of 17% since 1996. It was the top source region of recent immigrants, comprising 61.8% of all newcomers. Africa ranked second as a source region of recent immigrants to Canada with 13.4% in 2016. The following Muslim‐majority countries were among the top countries of birth of recent immigrants: Nigeria, Algeria, Egypt, Morocco, Iran, Pakistan, Syria, and Iraq.^9^

Cancer screening literature that focusses on disparities for immigrants has highlighted a number of individual and system level factors associated with lower screening uptake such as limited economic power, language barriers, immigration status (economic, family, refugee and other), world region of birth, limited knowledge about the benefits of preventive health care, lack of a primary care provider, not being enrolled in primary care practice, having a male or internationally trained physician, and inflexible clinic hours as being.^7^, ^10^, ^11^, ^12^, ^13^, ^14^, ^15^ However, there is a dearth of knowledge related to the role of religious affiliation in screening uptake for immigrants. It is suggested that religion affiliation is one of the health‐related factors that cuts across and often unites different racial, ethnic, and socioeconomic categories. Religion plays a significant role in people's understanding of their disease, health behavior, health service utilization, and compliance with medical recommendations.^16^, ^17^, ^18^, ^19^

We have previously examined cervical and breast cancer screening for women from Muslim‐majority countries, and found differences in screening uptake.^20^, ^21^

Considering a higher influx of immigrants from Muslim‐majority countries from Asia and from Africa, and the fact that approximately 73% of Canadian Muslims live in Ontario, the main goal of this study was to examine the CRC screening uptake (ie, FOBT modality) and its predictors among immigrant Muslim and non‐Muslim majority countries residing in Ontario using country of birth and region of origin as a proxy for religious affiliation. The findings will help to understand the role of religious affiliation in cancer screening uptake.

## 2 METHODS

### 2.1 Study design

We conducted a population‐based retrospective cohort study using multiple linked healthcare administrative databases at ICES, an independent nonprofit provincial research organization that houses population‐based health and administrative data. Databases at ICES were linked using unique encoded identifiers. This study falls under Section 45 of Ontario's Personal Health Information Protection Act which does not require Research Ethics Board Review. Section 45 authorizes health information custodians to disclose personal health information to a prescribed entity, like ICES, without consent.

### 2.2 Study population

Our study cohort was created by linking the Registered Persons Database (RPDB) and the Immigration, Refugees and Citizenship Canada database (IRCC) and included immigrant people aged 50‐74, who were eligible for the universal Ontario Health Insurance Plan (OHIP). The RPDB database contains the age, sex, and postal code of all Ontario residents who are eligible for Ontario's health insurance plan that covers healthcare expenses of all permanent residents and certain refugees. The IRCC database comprises demographic characteristics of landed immigrants and refugees in Canada since 1985.

#### 2.2.1 Inclusion and exclusion criteria

The inclusion criteria consisted of people who: (a) were alive and eligible for health care coverage for the entire study period between 1 April 2013 and 31 March 2015; (b) were in the 50‐74 year age range during the study period; and (c) resided in Ontario. The targeted 50‐74 age group and 2‐year study period correspond to the recommended time line and eligibility criteria for FOBT as recommended by the CCC screening program.

The exclusion criteria were people who (a) had a diagnosis of colorectal cancer or inflammatory bowel disease; (b) died prior to 31 March 2015; (c) were not continuously eligible for OHIP; and (d) did not have a documented country of birth.

**Figure 1:**
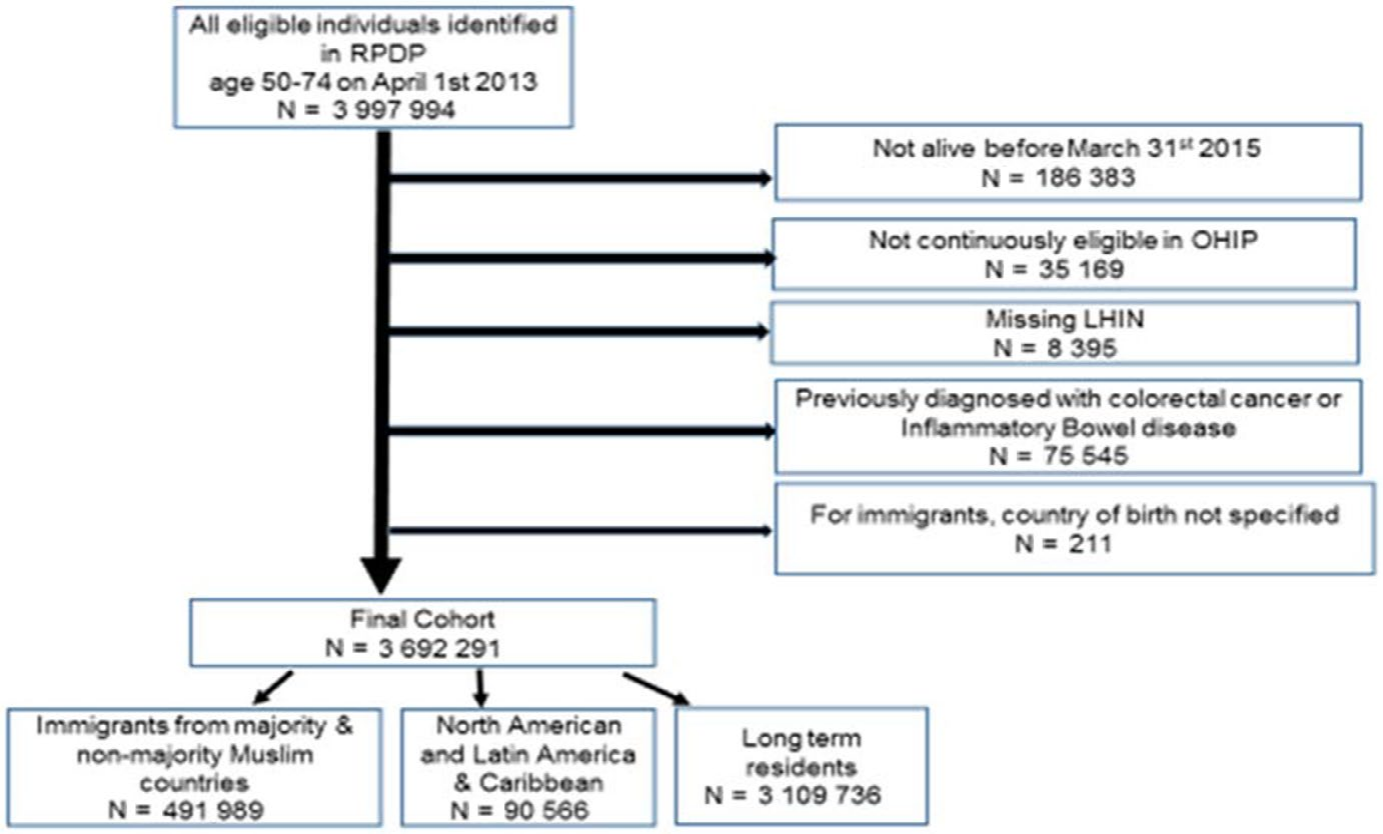
Creation of study cohort eligible for colorectal screening (FOBT) in Ontario, Canada

Figure 1 shows the creation of the final study cohort which included 3 692 291 people who met our inclusion and exclusion criteria. Of those, 582 555 were immigrants and 3 109 736 were Canadian long‐term residents (women and men who were either born in Canada or immigrated prior to 1985). Immigrants' country of birth was used for their classification into Muslim‐majority (ie, 50% or more of the country's estimated 2010 population identifying themselves as Muslim) or non‐Muslim majority using data from the Pew Research Center (Appendix S1). Of the 582 555 immigrants in our cohort, we excluded 90 566 immigrants from Latin America as well as the Caribbean and North America regions as Muslim‐majority countries in those regions are nonexistent. Immigrants from Muslim and non‐Muslim majority countries (n = 491 989) were then grouped by their region of birth using a previously published modified version of the World Bank classification system. This resulted in the following five regions: (a) South Asia, (b) the Middle East and North Africa, (c) Eastern Europe and Central Asia, (d) Sub‐Saharan Africa, (e) East Asia and Pacific).

### 2.3 Outcome measures

Our dichotomous study outcome was adherence to FOBT screening during the study period (1 April 2013 to 31 March 2015). Physician claims submitted to OHIP were used to identify people who had undergone FOBT. The OHIP database includes billing and diagnostic information submitted by approximately 95% of Ontario's physicians.

### 2.4 Study variables and associated databases

The analysis included the following individual and system level variables that we hypothesized could influence the relationship between Muslim religion and FOBT use: age, sex, immigration class (economic, family, refugee, or other), language ability (English, French, both or neither), neighborhood income (categorized into five quintiles—Q1 (lowest income) to Q5 (highest income), gender and location of training of the primary care physician (ie, graduation from Canadian or foreign medical schools), and type of primary care patient enrollment model (PEM) (ie, Family Health Group [FHG]—Primarily fee‐for‐service model, Comprehensive Care Model [CCM]—Primarily fee‐for‐service model, Family Health Organizations [FHO]—Primarily capitation model #1, Family Health Networks [FHN]—Primarily capitation model #2). Attachment to a primary care provider was assessed using data from the Client Agency Program Enrolment (CAPE) and the Corporate Providers' Databases which capture all Ontarians who are enrolled with a physician in a patient enrollment model (PEM). PEMs include alternate models of primary care delivery and physician disbursement ranging from independent physicians to group‐based multidisciplinary practices, capitation‐based blended payment to fee‐for‐service‐based blended payment and/or salary‐based blended payment with further financial incentives for promotion of cancer screening.^22^, ^23^ The ICES Physician Database (IPDB) was used to capture demographic information of physicians (ie, gender, training). The 2006 Canadian Census and RPDB were used to assess sociodemographic data of the study participants (ie, neighborhood income quintile and language ability). (For more details regarding databases, please see Appendix S2.)

### 2.5 Statistical analysis

We used descriptive statistics to describe the study population by world region of origin and by Muslim and non‐Muslim‐ majority countries. Inferential analyses including both bivariate and multivariate Poisson with robust error variance were applied to examine FOBT use disparities between Muslim and non‐Muslim immigrants stratified by their region of origin, and to identify the predictors of nonadherence to CRC screening based on our study variables noted above. Region of origin was included as a variable in the overall regression model to diminish unmeasured confounders that might exist when comparing people from different regions of the world with different cultures and economies. We controlled for all study variables noted above. All statistical tests were performed using SAS version 9.4 (SAS Institute) and set at the two‐sided 5% level of significance.

## 3 RESULTS

The sociodemographic and clinical characteristics of Ontario immigrants aged 50‐74 years who were eligible for CRC screening (FOBT) are displayed in Table 1 by region of origin and Muslim majority countries. The most common source region for immigrants in our cohort was East Asia and Pacific (33%), followed by South Asia (26%), Europe and Central Asia (24%), the Middle East and North Africa (10%), and Sub‐Saharan Africa (6%). Approximately 20% of immigrants were from Muslim majority countries, and of those the majority came from the Middle East and North Africa, and South Asia.

**Table 1:** Descriptive characteristics of immigrants eligible for FOBT in Ontario

Variable	South Asia		Middle East and North Africa		Europe and Central Asia		Sub‐Saharan Africa		East Asia and Pacific		Ontario long‐term residents
	Muslim majority	Non‐Muslim majority	Muslim majority	Non‐Muslim majority	Muslim majority	Non‐Muslim majority	Muslim majority	Non‐Muslim majority	Muslim majority	Non‐Muslim majority	
	N = 34 785	N = 93 835	N = 47 718	N = 1572	N = 7140	N = 114 372	N = 6343	N = 23 400	N = 3 039	N = 159 785	N = 3 109 736
Age at 1 April 2013											
Mean ± SD	57.03 ± 5.65	59.21 ± 6.49	57.56 ± 6.10	58.45 ± 6.25	56.85 ± 5.88	57.41 ± 5.80	56.80 ± 5.73	56.57 ± 5.56	57.65 ± 6.26	57.64 ± 6.16	59.58 ± 6.38
Median (IQR)	56 (52‐60)	58 (53‐65)	56 (52‐62)	57 (53‐63)	55 (52‐60)	56 (53‐61)	55 (52‐60)	55 (52‐60)	56 (52‐62)	56 (52‐62)	59 (54‐65)
Income quintile, n (%)^b^, ^b^											
1—Low	10 734 (30.9%)	22 347 (23.8%)	10 597 (22.2%)	244 (15.5%)	1931 (27.0%)	23 364 (20.4%)	3393 (53.5%)	7328 (31.3%)	528 (17.4%)	35 972 (22.5%)	504 929 (16.2%)
2	7127 (20.5%)	25 063 (26.7%)	8207 (17.2%)	236‐240^a^, ^a^	1415 (19.8%)	21 844 (19.1%)	1173 (18.5%)	4745 (20.3%)	641‐645^a^, ^a^	38 891 (24.3%)	579 138 (18.6%)
3	7071 (20.3%)	23 811 (25.4%)	9703 (20.3%)	236 (15.0%)	1384 (19.4%)	22 718 (19.9%)	786 (12.4%)	4235 (18.1%)	614 (20.2%)	32 548 (20.4%)	607 377 (19.5%)
4	6674 (19.2%)	14 964 (15.9%)	10 763 (22.6%)	389 (24.7%)	1456 (20.4%)	25 696 (22.5%)	668 (10.5%)	3876 (16.6%)	733 (24.1%)	30 155 (18.9%)	673 149 (21.6%)
5—High	3153 (9.1%)	7593 (8.1%)	8351 (17.5%)	464 (29.5%)	940 (13.2%)	20 586 (18.0%)	313 (4.9%)	3168 (13.5%)	520 (17.1%)	21 798 (13.6%)	734 310 (23.6%)
Missing	26 (0.1%)	57 (0.1%)	97 (0.2%)	1‐5^a^, ^a^	14 (0.2%)	164 (0.1%)	10 (0.2%)	48 (0.2%)	1‐5^a^, ^a^	421 (0.3%)	10 833 (0.3%)
Residence, n (%)											
Urban	34 686 (99.7%)	93 511 (99.7%)	47 570 (99.7%)	1556‐1560^a^, ^a^	7104 (99.5%)	109 746 (96.0%)	6336‐6340^a^, ^a^	23 185 (99.1%)	3018 (99.3%)	158 911 (99.5%)	2 624 707 (84.4%)
Rural	99 (0.3%)	324 (0.3%)	148 (0.3%)	11‐15^a^, ^a^	36 (0.5%)	4626 (4.0%)	1‐5^a^, ^a^	215 (0.9%)	21 (0.7%)	874 (0.5%)	485 029 (15.6%)
Language ability, n (%)											
English	24 644 (70.8%)	58 129 (61.9%)	29 649 (62.1%)	1245 (79.2%)	3715 (52.0%)	53 782 (47.0%)	4873 (76.8%)	18 586 (79.4%)	2548 (83.8%)	95 396 (59.7%)	—
French	36 (0.1%)	86‐90^a^, ^a^	1096‐1100^a^, ^a^	9 (0.6%)	76‐80^a^, ^a^	1296‐1300^a^, ^a^	96‐100^a^, ^a^	816‐820^a^, ^a^	1‐5^a^, ^a^	356‐360^a^, ^a^	—
Both	215 (0.6%)	465 (0.5%)	3312 (6.9%)	78 (5.0%)	234 (3.3%)	5797 (5.1%)	156 (2.5%)	1935 (8.3%)	21‐25^a^, ^a^	674 (0.4%)	—
Neither	9890 (28.4%)	35 151 (37.5%)	13 655 (28.6%)	240 (15.3%)	3110 (43.6%)	53 480 (46.8%)	1214 (19.1%)	2058 (8.8%)	464 (15.3%)	63 356 (39.7%)	—
Missing	0 (0.0%)	1‐5^a^, ^a^	1‐5^a^, ^a^	0 (0.0%)	1‐5^a^, ^a^	1‐5^a^, ^a^	1‐5^a^, ^a^	1‐5^a^, ^a^	0 (0.0%)	1‐5^a^, ^a^	—
Immigrant class, n (%)											
Economic	19 788 (56.9%)	36 640 (39.0%)	25 811 (54.1%)	1034 (65.8%)	3828 (53.6%)	56 051 (49.0%)	996 (15.7%)	10 933 (46.7%)	1839 (60.5%)	86 202 (53.9%)	—
Family	6791 (19.5%)	42 789 (45.6%)	8072 (16.9%)	391 (24.9%)	1703 (23.9%)	27 719 (24.2%)	824 (13.0%)	4871 (20.8%)	982 (32.3%)	49 380 (30.9%)	—
Refugee	498 (1.4%)	1708 (1.8%)	380 (0.8%)	1‐5^a^, ^a^	159 (2.2%)	431 (0.4%)	131‐135^a^, ^a^	257 (1.1%)	11 (0.4%)	1103 (0.7%)	—
Other	7362 (21.2%)	12 559 (13.4%)	12 633 (26.5%)	136‐140^a^, ^a^	1409 (19.7%)	30 084 (26.3%)	4388 (69.2%)	7293 (31.2%)	189 (6.2%)	20 276 (12.7%)	—
Missing	346 (1.0%)	139 (0.1%)	822 (1.7%)	7 (0.4%)	41 (0.6%)	87 (0.1%)	1‐5^a^, ^a^	46 (0.2%)	18 (0.6%)	2824 (1.8%)	—
Resource Utilization Bands (RUBs, n (%)^c^											
0	6044 (17.4%)	12 691 (13.5%)	8862 (18.6%)	317 (20.2%)	1203 (16.8%)	17 389 (15.2%)	1347 (21.2%)	3248 (13.9%)	521		
(17.1%)	28 790 (18.0%)	263 591 (8.5%)									
1	521 (1.5%)	1420 (1.5%)	940 (2.0%)	47 (3.0%)	187 (2.6%)	3952 (3.5%)	175 (2.8%)	593 (2.5%)	89 (2.9%)	4083 (2.6%)	101 425 (3.3%)
2	2897 (8.3%)	8505 (9.1%)	4441 (9.3%)	125 (8.0%)	806 (11.3%)	15 191 (13.3%)	639 (10.1%)	2759 (11.8%)	393 (12.9%)	20 893 (13.1%)	411 396 (13.2%)
3	18 875 (54.3%)	54 944 (58.6%)	24 128 (50.6%)	767 (48.8%)	3823 (53.5%)	60 288 (52.7%)	3048 (48.1%)	13 102 (56.0%)	1661 (54.7%)	87 424 (54.7%)	1 731 217 (55.7%)
4+	6448 (18.5%)	16 275 (17.3%)	9347 (19.6%)	316 (20.1%)	1 121 (15.7%)	17 552 (15.3%)	1134 (17.9%)	3698 (15.8%)	375 (12.3%)	18 595 (11.6%)	602 107 (19.4%)
Enrollment model, n (%)											
Primarily fee‐for‐service(FHG/CCM)	20 481 (58.9%)	56 309 (60.0%)	23 439 (49.1%)	662 (42.1%)	3450 (48.3%)	44 239 (38.7%)	2744 (43.3%)	10 917 (46.7%)	1656 (54.5%)	91 846 (57.5%)	931 652 (30.0%)
Primarily Caption model #1 (FHO)	6435 (18.5%)	19 213 (20.5%)	12 787 (26.8%)	583 (37.1%)	2011 (28.2%)	41 949 (36.7%)	1739 (27.4%)	7589 (32.4%)	719 (23.7%)	32 194 (20.1%)	1 600 891 (51.5%)
Primarily Caption model #2 (FHN)	23 (0.1%)	92 (0.1%)	100 (0.2%)	1‐5^a^, ^a^	14 (0.2%)	928 (0.8%)	6‐10^a^, ^a^	117 (0.5%)	8 (0.3%)	248 (0.2%)	101 796 (3.3%)
No primary care	5368 (15.4%)	10 921 (11.6%)	7783 (16.3%)	249 (15.8%)	1053 (14.7%)	14 663 (12.8%)	1242 (19.6%)	2871 (12.3%)	420 (13.8%)	23 084 (14.4%)	232 804 (7.5%)
Traditional fee ‐for service	2400 (6.9%)	7058 (7.5%)	3477 (7.3%)	70 (4.5%)	595 (8.3%)	11 977 (10.5%)	603 (9.5%)	1770 (7.6%)	200 (6.6%)	11 194 (7.0%)	165 644 (5.3%)
Other model	78 (0.2%)	242 (0.3%)	132 (0.3%)	1‐5^a^, ^a^	17 (0.2%)	616 (0.5%)	6‐10^a^, ^a^	136 (0.6%)	36 (1.2%)	1219 (0.8%)	76 949 (2.5%)
Physician sex, n (%)											
Female	12 399 (35.6%)	27 378 (29.2%)	13 360 (28.0%)	398 (25.3%)	2049 (28.7%)	39 681 (34.7%)	1183 (18.7%)	5840 (25.0%)	895 (29.5%)	39 240 (24.6%)	921 593 (29.6%)
Male	16 902 (48.6%)	55 338 (59.0%)	26 444 (55.4%)	921 (58.6%)	3998 (56.0%)	59 560 (52.1%)	3894 (61.4%)	14 539 (62.1%)	1714 (56.4%)	96 556 (60.4%)	1 939 312 (62.4%)
Missing	5484 (15.8%)	11 119 (11.8%)	7914 (16.6%)	253 (16.1%)	1093 (15.3%)	15 131 (13.2%)	1266 (20.0%)	3021 (12.9%)	430 (14.1%)	23 989 (15.0%)	248 831 (8.0%)
Physician training, n (%)											
International	18 882 (54.3%)	59 257 (63.2%)	25 120 (52.6%)	392 (24.9%)	3197 (44.8%)	51 996 (45.5%)	2573 (40.6%)	9119 (39.0%)	976 (32.1%)	54 838 (34.3%)	707 623 (22.8%)
Domestic	10 419 (30.0%)	23 459 (25.0%)	14 684 (30.8%)	927 (59.0%)	2850 (39.9%)	47 245 (41.3%)	2504 (39.5%)	11 260 (48.1%)	1633 (53.7%)	80 958 (50.7%)	2 153 282 (69.2%)
Missing	5484 (15.8%)	11 119 (11.8%)	7914 (16.6%)	253 (16.1%)	1093 (15.3%)	15 131 (13.2%)	1266 (20.0%)	3021 (12.9%)	430 (14.1%)	23 989 (15.0%)	248 831 (8.0%)

^a^ Exact counts suppressed for privacy reasons in at least one of the cells.

^b^ An income quintile is a measure of neighborhood socioeconomic status that divides the population into five income groups (from lowest income to highest income) so that approximately 20% of the population is in each group. These are based on methods developed at Statistic Canada. Quintiles are based on the average income per single‐person equivalent in a dissemination area obtained from the Canadian Census, and also incorporate size of geographic area of residence.

^c^ RUBs = Resource Utilization Bands are part of the Johns Hopkins Adjusted Clinical Group® (ACG®) Case Mix System. The RUBs are used to categorize patients based on their expected use of health care resources and range from 0 (lowest expected health care costs) to 5 (highest expected health care costs). 0—Nonuser, 1—healthy user, 2—low morbidity, 3—moderate morbidity, 4—high morbidity, 5—very high morbidity.

More than 50% of immigrants resided in the two lowest income quintile neighborhoods compared to Canadian long‐term residents (ie, approximately 35%), with those from Muslim‐majority countries being more likely to live in the lowest income quintile. Income variations existed across immigrants' region of origin with Sub‐Saharan Africans from Muslim Majority countries having the highest proportion of people living in the two lowest income quintiles (72%) followed by South Asia (51%), Europe and Central Asia (47%) and Middle Eastern and North African (39%). Less than 5% of immigrants resided in rural area compared to 16% of Canadian long‐term residents. The proportion of immigrants who could not converse in either English or French varied by the region of origin, ranging from 11% of Sub‐Saharan Africa population to 47% of Europe and Central Asia. Although, the median years since immigration to Canada varied from 13 to 18 years, a higher percentage of immigrants had no primary care provider compared to Canadian long‐term residents (13.8% vs 7.5%). The proportion of immigrants without a primary care provider was consistently higher among Muslim majority countries compared to non‐Muslim Majority countries across all the region of origins with the exception of immigrants from East Asia and Pacific for whom the proportion was approximately the same (ie, 14%). Similarly, the majority of immigrants from Muslim majority countries consulted an internationally trained physician across all the regions of origin with the exception of East Asia and Pacific. Male physicians were more likely to be the primary care providers for immigrants across all regions of origin. The proportion of Muslim and non‐Muslim immigrants across all regions of origin having no health care resource utilization (ie, RUB of 0) was approximately four times more than Canadian long‐term residents (ie, 31% to 39% across the region of origin compared to about 9% among Canadian long‐term residents).

**Figure 2:**
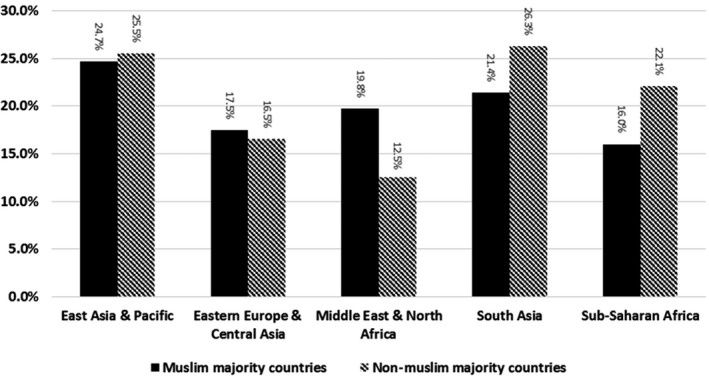
Proportion of Ontario immigrants screened with FOBT by region of origin and Muslim majority status

Figure 1 shows that approximately 78% of immigrants in our cohort were not up to date for FOBT. The highest FOBT screening rate was among immigrants from East Asia and Pacific (25.5%) followed by South Asia (24.9%) and Sub‐Saharan (20.8%). The lowest FOBT screening rate was among Eastern Europe and Central Asia (16.6%) followed by the Middle East and North Africa (19.5%). Although the proportion of screened was similar among Muslim and non‐Muslim majority from East Asia and Pacific, and Eastern Europe and Central Asia, the proportion of screened was significantly higher among non‐Muslim than Muslim majority countries in South Asia and Sub‐Saharan Africa but significantly lower among non‐Muslim than Muslim majority countries in the Middle East and North Africa (Figure 2).

Bivariate analyses by region of origin (Table 2) revealed higher likelihood of FOBT screening among those living in low income neighborhoods (eg, adjusted relative risk (ARR) 1.24 [95% confidence interval (CI) 1.14‐1.34] for immigrants from Sub‐Saharan Africa), having an internationally trained physician, and having been admitted to Canada under the family and refugee classes. However, having no primary care provider and having a male physician significantly reduced the likelihood of FOBT screening. For example, South Asian immigrants with no primary care provider had an ARR of 0.22 [95% CI 0.21‐0.23] versus those with either a female provider or who were in capitation model type #1.

**Table 2:** Bivariate analyses using Poisson (with robust error variance) where FOBT screening is represented by relative risks (95% confidence intervals)

Variables	South Asia	Middle East and North Africa	Eastern Europe and Central Asia	Sub‐Saharan Africa	East Asia and Pacific
Income quintile	RR (95% CI), *P*‐value	RR (95% CI), *P*‐value	RR (95% CI), *P*‐value	RR (95% CI), *P*‐value	RR (95% CI), *P*‐value
1 vs 5 (highest)	1.07 (1.03‐1.12) *P* = .0006	1.09 (1.03‐1.15) *P* = .0053	1.02 (0.97‐1.06) *P* = .4704	1.24 (1.14‐1.34) *P*<.0001	1.16 (1.12‐1.19) *P*<.0001
2 vs 5	1.15 (1.11‐1.20) *P*<.0001	1.07 (1.00‐1.14) *P* = .0419	1.14 (1.09‐1.19) *P*<.0001	1.29 (1.18‐1.40) *P*<.0001	1.20 (1.16‐1.23) *P*<.0001
3 vs 5	1.20 (1.16‐1.25) *P*<.0001	1.12 (1.06‐1.19) *P* = .0001	1.06 (1.02‐1.11) *P* = .0034	1.19 (1.09‐1.30) *P* = .0002	1.14 (1.10‐1.17) *P*<.0001
4 vs 5	1.11 (1.061.16) *P*<.0001	1.16 (1.09‐1.23) *P*<.0001	1.04 (1.00‐1.08) *P* = .0589	1.24 (1.14‐1.36) *P*<.0001	1.11 (1.08‐1.15) *P*<.0001
Immigrant class	RR (95% CI), *P*‐value	RR (95% CI), *P*‐value	RR (95% CI), *P*‐value	RR (95% CI), *P*‐value	RR (95% CI), *P*‐value
Family vs Economic	1.30 (1.271.33) *P*<.00011	1.18 (1.12‐1.23) *P*<.0001	1.06 (1.02‐1.09) *P* = .0005	1.17 (1.10‐1.24) *P*<.0001	1.36 (1.33‐1.38) *P*<.0001
Other vs Economic	1.22 (1.18‐1.25) *P*<.0001	1.18 (1.13‐1.23) *P*<.0001	0.88 (0.85‐0.91) *P*<.0001	1.10 (1.04‐1.15) *P* = .0004	1.31 (1.27‐1.34) *P*<.0001
Refugee vs Economic	1.38 (1.29‐1.47) *P*<.0001	1.24 (1.03‐1.50) *P* = .0226	0.93 (0.781.13) *P* = .4812	1.39 (1.17‐1.64) *P* = .0001	1.72 (1.59‐1.85) *P*<.0001
Language ability	RR (95% CI), *P*‐value	RR (95% CI), *P*‐value	RR (95% CI), *P*‐value	RR (95% CI), *P*‐value	RR (95% CI), *P*‐value
English vs both	0.98 (0.85‐1.12) *P* = .7181	1.07 (0.99‐1.15) *P* = .0902	0.98 (0.93‐1.04) *P* = .5576	0.87 (0.80‐0.94) *P* = .0007	0.99 (0.87‐1.13) *P* = .9073
French vs both	1.00 (0.71‐1.42) *P* = .9908	1.34 (1.18‐1.53) *P*<.0001	1.07 (0.94‐1.22) *P* = .2794	1.16 (1.02‐1.33) *P* = .0251	1.21 (0.98‐1.49) *P* = .0702
Neither vs both	1.22 (1.06‐1.40) *P* = .0046	1.25 (1.15‐1.35) *P*<.0001	1.03 (0.97‐1.10) *P* = .2653	0.93 (0.84‐1.03) *P* = .1404	1.19 (1.04‐1.36) *P* = .0115
Physician sex	RR (95% CI), *P*‐value	RR (95% CI), *P*‐value	RR (95% CI), *P*‐value	RR (95% CI), *P*‐value	RR (95% CI), *P*‐value
Male vs Female	0.93 (0.91‐0.95) *P*<.0001	0.90 (0.87‐0.94) *P*<.0001	0.92 (0.90‐0.94) *P*<.0001	0.90 (0.86‐0.95) *P*<.0001	0.95 (0.93‐0.97) *P*<.0001
Missing vs female	0.22 (0.21‐0.23) *P*<.0001	0.22 (0.20‐0.25) *P*<.0001	0.29 (0.27‐0.31) *P*<.0001	0.33 (0.300.37) *P*<.0001	0.25 (0.24‐0.26) *P*<.0001
Canadian medical graduate	RR (95% CI), *P*‐value	RR (95% CI), *P*‐value	RR (95% CI), *P*‐value	RR (95% CI), *P*‐value	RR (95% CI), *P*‐value
No vs Yes	1.26 (1.23‐1.29) *P*<.0001	1.22 (1.17‐1.27)p=<0.0001	0.93 (0.90‐0.95) *P*<.0001	1.06 (1.01‐1.11) *P* = .015	1.13 (1.11‐1.15) *P*<.0001
Missing vs Yes	0.27 (0.26‐0.29) *P*<.0001	0.27 (0.25‐0.30) *P*<.0001	0.93 (0.90‐0.95) *P*<.0001	0.37 (0.33‐0.41) *P*<.0001	0.27 (0.26‐0.28) *P*<.0001
Primary care model	RR (95% CI), *P*‐value	RR (95% CI), *P*‐value	RR (95% CI), *P*‐value	RR (95% CI), *P*‐value	RR (95% CI), *P*‐value
Primarily fee‐for‐service model vs primarily capitation model #1	1.04 (1.01‐1.06) *P* = .0015	1.09 (1.05‐1.13) *P*<.0001	0.79 (0.77‐0.81) *P*<.0001	1.04 (0.99‐1.09) *P* = .1096	1.08 (1.05‐1.10) *P*<.0001
Primarily capitation model #2 vs primarily capitation model #1	1.26 (0.98‐1.63) *P* = .0668	1.47 (1.11‐1.95) *P* = .0077	1.32 (1.19‐1.46) *P*<.0001	1.62 (1.28‐2.05) *P*<.0001	1.13 (0.94‐1.35) *P* = .2032
Other model vs primarily capitation model #1	1.18 (1.01‐1.39) *P* = .0398	1.09 (0.80‐1.48) *P* = .5776	1.13 (0.98‐1.30) *P* = .0917	1.23 (0.94‐1.61) *P* = .1238	1.42 (1.32‐1.52) *P*<.0001
Traditional fee‐for‐service vs primarily capitation model #1	0.77 (0.73‐0.80) *P*<.0001	0.79 (0.73‐0.86) *P*<.0001	0.56 (0.53‐0.59) *P*<.0001	0.81 (0.74‐0.89) *P*<.0001	0.75 (0.72‐0.78) *P*<.0001
No primary care vs primarily capitation model #1	0.22 (0.21‐0.23) *P*<.0001	0.23 (0.21‐0.26) *P*<.0001	0.25 (0.23‐0.26) *P*<.0001	0.34 (0.30‐0.38) *P*<.0001	0.24 (0.22‐0.25) *P*<.0001

Multivariate analyses (Table 3) show that both immigrant and nonimmigrant factors influence the observed disparity in screening uptake. Overall, being born in a Muslim‐majority country was significantly associated with lower FOBT uptake (ARR 0.92 [95% CI 0.90‐0.93]), as were having a male physician (adjusted relative risk 0.95 [95% CI 0.94‐0.96]), having no primary care physician (ARR 0.22[95% CI 0.21 −0.24]), and being in a traditional fee‐for service primary care model (ARR 0.68 [95% CI 0.66‐0.69]). There was variation in the association by region of origin. For example, FOBT uptake was lowest among people born in Sub‐Saharan Muslim majority countries vs non‐Muslim majority countries (ARR 0.73 [95% CI 0.68‐0.78]) and highest among people born in the Middle East and North Africa Muslim majority countries versus non‐Muslim majority countries (ARR 1.48[95% CI 1.29‐1.69]). The likelihood of FOBT uptake was significantly higher for those living in lowest income neighborhoods (adjusted relative risk 1.1 [95% CI 1.08‐0.13]), and having an internationally educated primary care physician (ARR 1.11 [95% CI 1.11‐1.13]). The likelihood of FOBT use was also associated with the region of origin. Immigrants from Europe and Central Asia (ARR 0.63 [95% CI 0.62‐0.964]), Sub‐Saharan Africa (0.84 [95% CI 0.82‐0.86]), the Middle East and North Africa (ARR 0.85 [95% CI 0.82‐0.87]) and South Asia (ARR 0.94 [95% CI 0.93‐0.95]) were more likely to have inadequate FOBT screening compared to people from East Asia and Pacific.

**Table 3:** Multivariable Poisson regression with robust error variance, where adjusted relative risks represent FOBT uptake. All variables listed were included in analyses

Characteristics	South Asia				Middle East and North Africa				Europe and Central Asia				Sub‐Saharan Africa				East Asia and Pacific				Overall			
	RR	95% CI		*P*‐value	RR	95% CI		*P*‐value	RR	95% CI		*P*‐value	RR	95% CI		*P*‐value	RR	95% CI		*P*‐value	RR	95% CI		*P*‐value
Muslim majority vs non‐Muslim majority (reference)	0.88	0.86	0.9	<.0001	1.48	1.29	1.69	<.0001	1.1	1.0399	1.15	.0006	0.73	0.68	0.78	<.0001	1.02	0.95	1.08	.6359	0.92	0.9	0.93	<.0001
Age (years), continuous	1	1	1	.453	1	1	1	.6995	1	1.0023	1.01	<.0001	0.99	0.99	1	.0012	1	1	1	<.0001	1	1	1	.0014
Patient sex male vs female (reference)	0.95	0.93	0.97	<.0001	0.97	0.94	1.01	.1229	0.93	0.9017	0.95	<.0001	0.91	0.87	0.95	<.0001	0.95	0.93	0.97	<.0001	0.95	0.94	0.96	<.0001
Income quintile																								
1 v s5	1.08	1.04	1.12	.0001	1.1	1.03	1.17	.0022	1.08	1.0354	1.13	.0003	1.3	1.19	1.41	<.0001	1.1	1.07	1.13	<.0001	1.1	1.08	1.13	<.0001
2 vs 5	1.1	1.05	1.14	<.0001	1.07	1	1.14	.0406	1.17	1.1266	1.22	<.0001	1.3	1.19	1.42	<.0001	1.13	1.09	1.16	<.0001	1.13	1.11	1.16	<.0001
3 vs 5	1.13	1.08	1.17	<.0001	1.09	1.02	1.15	.0072	1.08	1.0363	1.13	.0003	1.18	1.07	1.29	.0005	1.08	1.05	1.11	<.0001	1.1	1.08	1.13	<.0001
4 vs 5	1.08	1.03	1.12	.0004	1.12	1.05	1.18	.0002	1.05	1.0095	1.1	.0157	1.23	1.12	1.35	<.0001	1.06	1.03	1.1	<.0001	1.08	1.06	1.1	<.0001
Physician sex																								
Male vs female	0.94	0.92	0.96	<.0001	0.93	0 9	0.97	.0004	0.93	0.9035	0.95	<.0001	0.91	0.87	0.96	.0003	0.96	0.94	0.98	<.0001	0.95	0.94	0.96	<.0001
Missing vs female	1.07	0.88	1.3	.5025	1.11	0.8	1.54	.5364	0.92	0.7434	1.13	.4123	0.86	0.64	1.15	.3211	1.15	1.04	1.28	.0078	1.07	0.98	1.15	.1138
Canadian Medical Graduate^a^, ^a^																								
Foreign educated medical graduate vs Canadian medical graduate^a^, ^a^ (reference)	1.2	1.17	1.22	<.0001	1.19	1.15	1.24	<.0001	0.97	0.9448	1	.0283	1.06	1.01	1.11	.0104	1.14	1.13	1.16	<.0001	1.11	1.1	1.13	<.0001
Immigration class																								
Family vs economic	1.11	1.08	1.14	<.0001	1.07	1.01	1.12	.0118	1.02	0.9903	1.06	.1747	1.16	1.09	1.23	<.0001	1.25	1.23	1.28	<.0001	1.16	1.15	1.18	<.0001
Other vs economic	1.07	1.04	1.10	<.0001	1.02	0.97	1.06	.44	0.87	0.84	0.90	<.0001	1.14	1.08	1.21	<.0001	1.20	1.17	1.23	<.0001	1.05	1.04	1.07	<.0001
Refugee dependent vs economic	1.09	1.02	1.17	.01	1.05	0.87	1.27	.59	0.88	0.73	1.06	.18	1.36	1.15	1.61	.00	1.44	1.34	1.56	<.0001	1.19	1.14	1.25	<.0001
Language ability																								
English vs both	0.94	0.82	1.07	.36	1.01	0.93	1.09	.87	0.97	0.91	1.03	.33	0.87	0.80	0.94	.00	0.93	0.82	1.06	.29	0.92	0.89	0.96	<.0001
French vs both	0.94	0.67	1.33	.74	1.23	1.09	1.40	.00	1.08	0.95	1.22	.24	1.10	0.96	1.26	.16	1.16	0.95	1.43	.14	1.14	1.06	1.21	.00
Neither vs both	1.02	0.89	1.17	.74	1.07	0.98	1.16	.12	1.04	0.98	1.10	.21	0.89	0.80	0.98	.02	1.08	0.95	1.23	.23	1.01	0.97	1.05	.65
Enrollment model																								
Primarily fee‐for‐service model (FHG/CCM) vs Primarily capitation model #l	0.98	0.96	1.01	.19	1.02	0.98	1.07	.25	0.80	0.78	0.82	<.0001	1.01	0.96	1.06	.64	1.04	1.02	1.06	.00	0.97	0.95	0.98	<.0001
Primarily capitation model #2 (FHN) vs Primarily capitation model #1	1.37	1.07	1.75	.01	1.53	1.16	2.02	.00	1.33	1.20	1.48	<.0001	1.44	1.14	1.82	.00	1.12	0.93	1.35	.22	1.39	1.29	1.51	<.0001
Traditional fee‐for‐service vs primarily capitation model #1	0.74	0.71	0.77	<.0001	0.75	0.69	0.81	<.0001	0.56	0.53	0.59	<.0001	0.80	0.73	0.88	<.0001	0.71	0.68	0.73	<.0001	0.68	0.66	0.69	<.0001
No primary care vs primarily capitation model #1	0.22	0.18	0.27	<.0001	0.22	0.16	0.31	<.0001	0.26	0.20	0.32	<.0001	0.38	0.28	0.52	<.0001	0.21	0.19	0.23	<.0001	0.22	0.21	0.24	<.0001
Other model vs primarily capitation model #1	1.15	0.98	1.35	.08	1.07	0.79	1.46	.66	1.12	0.97	1.29	.12	1.10	0.84	1.43	.49	1.34	1.24	1.44	<.0001	1.24	1.17	1.31	<.0001
World Region^b^, ^b^																								
Europe and Central Asia vs East Asia and Pacific																					0.63	0.62	0.64	<.0001
Middle East and North Africa vs East Asia and Pacific																					0.85	0.82	0.87	<.0001
South Asia vs East Asia and Pacific																					0.94	0.93	0.95	<.0001
Sub‐Saharan Africa vs East Asia and Pacific																					0.84	0.82	0.86	<.0001

a No missing category for Canadian Medical Graduates (CMG). Missing values for CMG and physician sex were identical as they are the result of linkage to the physician database; as such only estimates for one can be generated in the multivariate model.

b Only included in overall model.

## 4 DISCUSSION

We have shown that Canadian immigrants from Muslim‐majority countries have lower FOBT uptake than those from non‐Muslim majority countries. We have also shown that there is variation in FOBT use between Ontario immigrants from Muslim‐majority countries versus nonmajority countries across all five regions of origin (ie, South Asia, the Middle East and North Africa, Eastern Europe and Central Asia, Sub‐Saharan Africa, and East Asia and Pacific). For instance, people from Muslim‐majority countries in the Middle East and North Africa and Eastern Europe and Central Asia had relatively higher FOBT use than their peers from nonmajority countries. However, FOBT use was similar for majority and nonmajority countries in East Asia, and relatively lower for those from Muslim majority countries in South Asia and Sub‐Saharan Africa. These findings not only substantiate earlier studies^10^, ^11^, ^13^, ^14^, ^24^ by demonstrating screening disparities among the immigrant population but also add to the body of knowledge by exploring an underresearched area, namely religious affiliation as a contributing factor to cancer screening uptake disparities.

Our findings illustrate religion affiliation as an independent factor in shaping people's uptake of FOBT screening. However, the variation in screening uptake across region of origins further suggests the moderating influence of cultural norms, values and practices. Kleinman (1980)^25^ argues, that health experiences are shaped by cultural factors that govern how people perceive, evaluate and undertake medical treatment or preventive health measures. According to Kleinman, religions further contribute to cultural construction of clinical reality by guiding people's choices regarding when, how, and whom to seek help. Cultural norms and values in conjunction with religious teaching and practices form a unique set of rules that can direct people's health‐related decision‐making. For instance, concerns over modesty may prevent some Muslim patients from receiving medical treatment they would otherwise accept. Several studies reported that both Muslim men and women may refrain from disclosing health information during gender‐discordant medical encounters.^15^, ^17^, ^18^, ^26^, ^27^ Furthermore according to Fiqh (Islamic Jurisprudence‐Sharia‐instructions for Muslim conduct in daily life) cleanliness must be highly upheld with respect to urination and defecation. A Muslim's home is considered a place of worship and prayer. Contamination with feces in places where Muslims pray can violate the principle of cleanliness that is required for the worship. Anxiety regarding the collection of feces may be a barrier to screening uptake. This issue has not been explored and further research is needed.

The current Canadian immigration landscape includes migrants from source countries who experience economic, political, or religious conflicts both pre‐ and postmigration. Settlement issues such as shelter, employment, education, language, safety, food, social support, and other basic necessities of life may take priority over preventive health measures such as cancer screening. Decline in social class status through deskilling, underemployment as well as overt and covert racial and religious discrimination have been associated with mental stress and poor health in the immigrant communities.^28^, ^29^ Although it is not possible for us to determine these elements from this study, further qualitative studies are needed to explore such factors as potential barriers in uptake of CRC screening among immigrants from Muslim‐majority countries.

Interestingly, our study also found higher adherence to FOBT among those from low versus high income neighborhoods. This does not substantiate other studies that found a direct association between income and adherence to CRC screening.^13^, ^30^, ^31^, ^32^ This may be due to the fact that previous studies explored both FOBT and colonoscopy as CRC screening as opposed to our study which only focused on FOBT. However, our result is consistent with a recent study by Kiran et al^33^ in Ontario that found, although there was little difference in FOBT rates among immigrants by income quintile, the lowest FOBT rates were among those in the highest income quintile. In this same study, the colonoscopy rate was highest among those residing in high income neighborhoods. These discrepancies may be related to affluent patients having better negotiation skills and more power to exert their preferences for colonoscopy during the medical encounter. Research is needed to explain these income‐related CRC screening modality disparities.

Our results show an association between FOBT screening uptake and having a female primary care provider and being rostered in a PEM. These findings are consistent with prior research that demonstrated that having a regular primary care provider and certain characteristics of the primary care provider like gender, location of training, number of years in practice‐ promote cancer screening.^10^, ^11^, ^13^, ^34^, ^35^, ^36^ Hence, ensuring adequate access to primary care providers, particularly female providers for immigrants from Muslim Majority countries is necessary. Although we did not explore the congruency between the region of origin of patients and their physicians, our analyses showed that having an international medical graduate (IMG) promoted rather than hindering participation in CRC screening‐FOBT which is contrary to earlier findings.^7^, ^10^, ^11^, ^13^, ^14^, ^34^ Possible explanations may be either that IMGs training placed a high priority on CRC screening using FOBT specifically or years of practice which may have changed IMGs attitudes toward FOBT screening. Our study did not explore these elements and they warrant further examination. Our results also suggest that there is a need for physician‐targeted cancer screening outreach for male and non‐PEM physicians where the rate of FOBT screening is low. It is also feasible that these physician groups may preferentially offer colonoscopy to their patients.

Finally, although information regarding CRC screening and the importance of early detection is available online^5^, and although multilingual instructions and fact sheets regarding CRC screening have been developed by the Ontario ColonCancerCheck program,^37^ their usage is unknown. The mere translation of information does not necessarily guarantee the cultural and religious sensitivity and appropriateness of the information. It is hence imperative to ensure that collaboration and input of faith organizations and immigration and community‐based organizations are sought in the development of cancer screening instructions and fact sheets. Several studies have found that ethnic minority groups including Muslims often prefer verbal information that is delivered through face‐to‐face and other interactive approaches to help raise awareness about the availability and purpose of CRC screening^38^, ^39^, ^40^, ^41^

Our reliance on administrative data imposes several limitations on our findings: First, the use of country of birth as a proxy for religion may present a challenge since being born in a Muslim majority country does not indicate the religion of an individual or their religiosity. However, this was the best proxy available to use since data on religion and ethnicity are not captured in Ontario's health or administrative databases. Second, the IRCC data only contain information on immigrants who landed in Ontario since 1985. Those who landed in other provinces and then relocated to Ontario are not captured in IRCC, and would inadvertently not be included in our study cohort. Third, our study did not include colonoscopy utilization as part of our study outcome. Some of the study participants who were not up to date with FOBT may be up to date with colonoscopy. However, colonoscopy is not the recommended screening option in Ontario for the average‐risk population, and we have been careful to only draw conclusions about FOBT uptake, not CRC screening uptake, from our results. As well, our findings are in line with those of Shen et al^13^ who found similar patterns for region of origin and adherence to CRC screening. Fourth, the information related to the religion of Canadian‐born and long‐term residents of the province as well as family history of CRC were not available from our linked administrative databases. Fifth, we did not explore the role of other factors important in acculturation, such as education, length of residence in Canada, and the last country of residence prior to immigration to Canada (ie secondary migration). Sixth, we did not explore the following characteristics of primary care providers: duration of independent practice in Ontario, time since graduation, or whether world region of training was the same as patient's region of birth which could have provided a more comprehensive picture of cancer screening barriers and facilitators. Seventh, our study only focused on Muslims and excluded other religions, such as Christianity, Judaism, and Buddhism. Future research could explore similar health service research questions for other religious groups. However, this would best be facilitated with capturing religion affiliation in demographic databases on a broad scale in Ontario. Finally, the data did not allow the exploration of the role of structural variables such as unemployment, housing, transportation etc in uptake of FOBT screening among immigrants. Future qualitative studies are warranted to examine this issue.

## 5 CONCLUSIONS

In this population‐based study, we examined factors associated with nonadherence to FOBT screening among immigrants from Muslim majority countries. FOBT uptake was associated with world region of origin, immigration class, rostering in a PEM, providers' sex and training. Although low FOBT screening uptake exists across all the regions, immigrants from the Middle East and North Africa and Eastern Europe and Central Asia countries were the least likely to use FOBT. Attachment to primary care models, having a female primary care provider and having an internationally trained physician were associated with higher FOBT use among immigrants from Muslim majority countries. Our findings have several implications as possible strategies that could improve uptake of FOBT: (a) enhancing immigrants access to regular primary care providers, particularly female providers and enrollment in primary care models; (b) physician‐targeted cancer screening outreach particularly for Canadian trained males and non‐PEM (c) developing culturally sensitive and appropriate educational materials in collaboration with faith organizations and immigration and community‐based organizations to promote knowledge and awareness of immigrants regarding the availability and purpose of CRC screening; and finally 4) using interactive approaches (like face‐to‐face) in communication of CRC screening information among immigrants.

## CONFLICT OF INTEREST

The authors declare that they have no competing interests.

## AUTHORS’ CONTRIBUTIONS

Lisa Ellison and Erin Graves extracted the data based on specification provided by Mandana Vahabi and Aisha Lofters. Mandana Vahabi processed and analyzed the data and drafted the article; Aisha Lofters, Josephine Wong, Erin Graves, Cynthia Damba, and Richard Glazier reviewed the article critically for intellectual content. All the authors gave final approval of the version to be published and agreed to serve as guarantors of the work.

## ETHICS APPROVAL AND CONSENT TO PARTICIPATE

This study falls under Section 45 of Ontario's Personal Health Information Protection Act (PHIPA) which does not require Research Ethics Board Review. Section 45 authorizes health information custodians to disclose personal health information to a prescribed entity, like ICES, without consent.

## CONSENT FOR PUBLICATION

Not Applicable.

## Data Availability

All data generated or analyzed during this study are included in this published article [and its supplementary information files].

## References

[1] Cancer Canadian Society's Advisory Committee on Cancer Statistics [Internet] . Canadian cancer statistics. Toronto: Canadian Cancer Society, 2017 http://www.cancer.ca/Canadian-Cancer-Statistics-2017-EN. Accessed August 5, 2018.

[2] Health Public. Agency of Canada . Canadian cancer statistics, 2017 https://www.canada.ca/en/public-health/services/chronic-diseases/cancer/colorectal-cancer.html. Accessed August 5, 2018.

[3] Hewitson P , Glasziou P , Watson E , Towler B , Irwig L . Cochrane systematic review of colorectal cancer screening using the fecal occult blood test (hemoccult): an update. Am J Gastroenterol. 2008;103(6):1541‐1549. 18479499 10.1111/j.1572-0241.2008.01875.x

[4] Schoen RE , Pinsky PF , Weissfeld JL , et al. Colorectal‐cancer incidence and mortality with screening flexible sigmoidoscopy. New Engl J Med. 2012, 366(25), 2345‐2357. 22612596 10.1056/NEJMoa1114635 PMC3641846

[5] Cancer Care Ontario . Fecal occult blood test 2016 https://www.cancercareontario.ca/en/types-of-cancer/colorectal/screening://www.cancercareontario.ca/en/types-of-cancer/colorectal/screening. Accessed August 5, 2018 .

[6] Cancer Quality Council of Ontario . Colorectal cancer screening participation 2017 http://www.csqi.on.ca/by_patient_journey/screening/colorectal_screening_participation://www.csqi.on.ca/by_patient_journey/screening/colorectal_screening_participation. Accessed August 5, 2018.

[7] Buchman S , Rozmovits L , Glazier RH . Equity and practice issues to colorectal cancer screening: mixed methods study. Can Fam Physician. 2016;62(4):e186‐e193. 27618142 PMC4830674

[8] Statistics Canada . (2011). Immigration and ethnocultural diversity in canada. National household survey. https://www12.statcan.gc.ca/nhs-enm/2011/as-sa/99-010-x/99-010-x2011001-eng.cfm. Accessed August 5, 2018.

[9] Statistics Canada . (2017). Ethnicity and Ehnocultural Diversity in Canada: Key Results from 2016 Census. https://www150.statcan.gc.ca/n1/daily-quotidien/171025/dq/171025b-eng.pdf. Accessed June 15, 2019.

[10] Vahabi M , Lofter A , Kumar M , Glazier R . Breast cancer screening disparities in Ontario. Canada. BMC Public Health. 2015;15:679‐691. 26194189 10.1186/s12889-015-2050-5 PMC4508905

[11] Vahabi M , Lofters A , Kumar M , Glazier RH . Breast cancer screening disparities among immigrant women by world region of origin: a population‐based study in Ontario, Canada. Cancer Med. 2016;5(7):1670‐1686. 27105926 10.1002/cam4.700 PMC4944895

[12] Vahabi M . Knowledge of breast cancer and screening practices among Iranian immigrant women in Toronto. J Community Health. 2011; 36(2):265‐273. 20812026 10.1007/s10900-010-9307-9

[13] Lofters AK , Hwang SW , Moineddin R , Glazier RH . Cervical cancer screening among urban immigrants by region of origin: a population‐based cohort study. Prev Med. 2010;51(6):509‐516. 20932995 10.1016/j.ypmed.2010.09.014

[14] Shen S , Lofters A , Tinmouth J , Paszat L , Rabeneck L , Glazier RH . Predictors of non‐adherence to colorectal cancer screening among immigrants to Ontario: a population‐based study. Prev Med. 2018;111:180‐189. 29548788 10.1016/j.ypmed.2018.03.002

[15] Saadi A , Bond B , Percac‐Lima S . Perspectives on preventive health care and barriers to breast cancer screening among Iraqi women refugees. J Immigr Minor Health. 2012;14(4):633‐639. 21901446 10.1007/s10903-011-9520-3

[16] Padela AI , Curlin FA . Religion and disparities: considering the influence of Islam on the health of American Muslims. J Relig Health. 2014;52:1333‐1345. 10.1007/s10943-012-9620-y 22653653

[17] Carroll J , Epstein R , Fiscella K , Volpe E , Diaz K , Omar S . Knowledge and beliefs about health promotion and preventive health care among somali women in the United States. Health Care Women Int. 2007;28(4):360‐380. 17454183 10.1080/07399330601179935

[18] Padela AI , Gunter K , Killawi A , Heisler M . Religious values and healthcare accommodations: voices from the American Muslim Community. J Gen Intern Med. 2012;27(6):708‐715. 22215274 10.1007/s11606-011-1965-5 PMC3358400

[19] Reitmanova S , Gustafson D . “They can't understand it”: maternity health and care needs of immigrant Muslim women in St. John's, Newfoundland. Matern Child Health J. 2008;12(1):101‐111. 17592762 10.1007/s10995-007-0213-4

[20] Vahabi M , Lofters A , Kim E , et al. Breast cancer screening among women from Muslim majority countries in Ontario, Canada. Prev Med. 2017;105:176‐183. 28916289 10.1016/j.ypmed.2017.09.008

[21] Lofters A , Vahabi M , Kim E , Ellison L , Graves E , Glazier RH . Cervical cancer screening among women from Muslim majority countries in Ontario, Canada. Cancer Epidemiol Biomark Prev. 2017;26(10):1493‐1499. 10.1158/1055-9965.EPI-17-0323 28939586

[22] Glazier RH , Redelmeier DA . Building the patient‐centered medical home in Ontario. JAMA. 2010;303(21):2186‐7. 20516421 10.1001/jama.2010.753

[23] Kiran T , Wilton AS , Moineddin R , Paszat L , Glazier RH . Effect of payment incentives on cancer screening in Ontario primary care. Ann Fam Med. 2014;12(4):317‐323. 25024239 10.1370/afm.1664 PMC4096468

[24] Sandy B , Rozmovits L , Glazier RH . Equity and practice issues in colorectal cancer screening: mixed‐methods study. Can Fam Physician. 2016;62(4):e186‐e193. 27618142 PMC4830674

[25] Kleinman A . Patients and healers in the context of culture: an exploration of the borderland between anthropology, medicine and psychology. Berkeley: University of California Press; 1980.

[26] Hammoud MM , White CB , Fetters MD . Opening cultural doors: providing culturally sensitive healthcare to Arab American and American Muslim patients. Am J Obstet Gynecol. 2005;193:1307‐1311. 16202719 10.1016/j.ajog.2005.06.065

[27] Salman KF . Health beliefs and practices related to cancer screening among Arab Muslim women in an urban community. Health Care Women Int. 2012;33(1):45‐74. 22150266 10.1080/07399332.2011.610536

[28] Asanin J , Wilson K . I spent nine years looking for a doctor: exploring access to health care among immigrants in Mississauga, Ontario, Canada. Soc Sci Med. 2008;6:1271‐1283. 10.1016/j.socscimed.2007.11.043 18194831

[29] Danso R , Grant M . Access to housing as an adaptive strategy for immigrant groups: Africans in calgary. Can Ethn Stud. 2000;32(3):19‐43.

[30] Ramji F , Cotterchio M , Manno M , Rabeneck L , Gallinger S . Association between subject factors and colorectal cancer screening participation in Ontario, Canada. Cancer Detection Prevention. 2005;29(3):221‐226. 15896925 10.1016/j.cdp.2005.04.001

[31] Singh SM , Paszat LF , Li C , He J , Vinden C , Rabeneck L . Association of socioeconomic status and receipt of colorectal cancer investigation: a population‐based retrospective cohort study. CMAJ. 2004;17(5):461‐465. 10.1503/cmaj.1031921 PMC514642 15337726

[32] Doubeni CA , Laiyemo AO , Reed G , Field TS , Fletcher RH . Socioeconomic and racial patterns of colorectal cancer screening among Medicare enrollees in 200 to 2005. Cancer Epidemiol Biomarkers Prev. 2009;18(8):2170‐2175. 19622721 10.1158/1055-9965.EPI-09-0104 PMC3018698

[33] Kiran T , Glazier RH , Moineddin R , Gu S , Wilton AS , Paszat L . The impact of a population‐based screening program on income‐ and immigration‐related disparities in colorectal cancer screening. Cancer Epidemiol Biomarkers Prevention. 2017;26(9):1401‐1410. 10.1158/1055-9965.EPI-17-0301 28626067

[34] Lofters AK , Ng R , Lobb R . Primary care physician characteristics associated with cancer screening: a retrospective cohort study in Ontario, Canada. Cancer Med. 2015;4(2):212‐223. 25430885 10.1002/cam4.358 PMC4329005

[35] Lurie N , Margolis KL , McGovern PG , Mink PJ , Slater JS . Why do patients of female physicians have higher rates of breast and cervical cancer screening? J Gen Intern Med. 1997;12(1):34‐43. 9034944 10.1046/j.1525-1497.1997.12102.x PMC1497051

[36] Franks P , Bertakis KD . Physician gender, patient gender, and primary care. J Women's Health. 2003;12(1):73‐80. 10.1089/154099903321154167 12639371

[37] Ontario ministry of health and long term care http://www.health.gov.on.ca/en/pro/programs/coloncancercheck/fobt_multilingual.aspx, Accessed August 5, 2018.

[38] Palmer CK , Thomas MC , McGregor LM , von Wagner C , Raine R . Understanding low colorectal cancer screening uptake in South Asian faith communities in England–a qualitative study. BMC Public Health. 2015;15:998‐1005. 26423750 10.1186/s12889-015-2334-9 PMC4589976

[39] Karbani G , Lim J , Hewison J , et al. Culture, attitude and knowledge about breast cancer and preventive measures: a qualitative study of South Asian breast cancer patients in the UK. Asian Pacific J Cancer Prev. 2011;12:1619‐1626. 22126509

[40] Thomas V , Saleem T , Abraham R . Barriers to effective uptake of cancer screening among black and minority ethnic groups. Int J Palliative Nursing. 2005;11:562‐571. 10.12968/ijpn.2005.11.11.20096 16471043

[41] Austin K , Power E , Solarin I , Atkin W , Wardle J , Robb K . Perceived barriers to flexible sigmoidoscopy screening for colorectal cancer among UK ethnic minority groups: a qualitative study. J Med Screen. 2009;16:174‐179. 20054091 10.1258/jms.2009.009080

