# Supplementary material for: Fecal occult blood test screening uptake among immigrants from Muslim majority countries: A retrospective cohort study in Ontario, Canada
Source: Cancer Med. 2019 Sep 30;8(16):7108–22. doi: 10.1002/cam4.2541 (PMC6853827; doi:10.1002/cam4.2541)
Supplement: Supplementary file 1 [file CAM4-8-7108-s001.docx]

**Appendix 1. Classification of countries by Muslim majority status.**

| **Country** | **Region** | **Majority Muslim (Yes/No)** |
| --- | --- | --- |
| American Samoa | East Asia & Pacific | No |
| Cambodia | East Asia & Pacific | No |
| China | East Asia & Pacific | No |
| Cook Islands | East Asia & Pacific | No |
| Fiji | East Asia & Pacific | No |
| French Polynesia | East Asia & Pacific | No |
| Guam | East Asia & Pacific | No |
| Hong Kong | East Asia & Pacific | No |
| Japan | East Asia & Pacific | No |
| Kiribati | East Asia & Pacific | No |
| Marshall Islands | East Asia & Pacific | No |
| Mongolia | East Asia & Pacific | No |
| Nauru | East Asia & Pacific | No |
| New Caledonia | East Asia & Pacific | No |
| Northern Mariana Islands | East Asia & Pacific | No |
| Palau | East Asia & Pacific | No |
| Papua New Guinea | East Asia & Pacific | No |
| Philippines | East Asia & Pacific | No |
| Samoa | East Asia & Pacific | No |
| Singapore | East Asia & Pacific | No |
| Taiwan | East Asia & Pacific | No |
| Thailand | East Asia & Pacific | No |
| Timor-Leste | East Asia & Pacific | No |
| Tonga | East Asia & Pacific | No |
| Vanuatu | East Asia & Pacific | No |
| Vietnam | East Asia & Pacific | No |
| Vietnam north | East Asia & Pacific | No |
| Brunei | East Asia & Pacific | Yes |
| Indonesia | East Asia & Pacific | Yes |
| Malaysia | East Asia & Pacific | Yes |
| Albania | Eastern Europe & Central Asia | Yes |
| Azerbaijan | Eastern Europe & Central Asia | Yes |
| [Kazakhstan](http://self.gutenberg.org/articles/Islam_in_Kazakhstan) | Eastern Europe & Central Asia | Yes |
| Kosovo | Eastern Europe & Central Asia | Yes |
| Kyrgyzstan | Eastern Europe & Central Asia | Yes |
| [Tajikistan](http://self.gutenberg.org/articles/Islam_in_Tajikistan) | Eastern Europe & Central Asia | Yes |
| Turkey | Eastern Europe & Central Asia | Yes |
| [Turkmenistan](http://self.gutenberg.org/articles/Islam_in_Turkmenistan) | Eastern Europe & Central Asia | Yes |
| [Uzbekistan](http://self.gutenberg.org/articles/Islam_in_Uzbekistan) | Eastern Europe & Central Asia | Yes |
| Israel | Middle East & North Africa | No |
| Algeria | Middle East & North Africa | Yes |
| Bahrain | Middle East & North Africa | Yes |
| Djibouti | Middle East & North Africa | Yes |
| Egypt | Middle East & North Africa | Yes |
| Iran | Middle East & North Africa | Yes |
| Iraq | Middle East & North Africa | Yes |
| Jordan | Middle East & North Africa | Yes |
| Kuwait | Middle East & North Africa | Yes |
| Lebanon | Middle East & North Africa | Yes |
| Libya | Middle East & North Africa | Yes |
| Morocco | Middle East & North Africa | Yes |
| Oman | Middle East & North Africa | Yes |
| Palestinian Territories | Middle East & North Africa | Yes |
| Qatar | Middle East & North Africa | Yes |
| Saudi Arabia | Middle East & North Africa | Yes |
| Syria | Middle East & North Africa | Yes |
| Tunisia | Middle East & North Africa | Yes |
| United Arab Emirates | Middle East & North Africa | Yes |
| [Western Sahara](http://self.gutenberg.org/articles/Islam_in_Western_Sahara) | Middle East & North Africa | Yes |
| [Yemen](http://self.gutenberg.org/articles/Islam_in_Yemen) | Middle East & North Africa | Yes |
| Yemen | Middle East & North Africa | Yes |
| Bhutan | South Asia | No |
| India | South Asia | No |
| Nepal | South Asia | No |
| Sri Lanka | South Asia | No |
| Afghanistan | South Asia | Yes |
| Bangladesh | South Asia | Yes |
| Maldives | South Asia | Yes |
| Pakistan | South Asia | Yes |
| Democratic Republic of Congo | Sub Saharan Africa | No |
| [Burkina Faso](http://self.gutenberg.org/articles/Islam_in_Burkina_Faso) | Sub Saharan Africa | Yes |
| Comoros | Sub Saharan Africa | Yes |
| Chad | Sub Saharan Africa | Yes |
| Gambia | Sub Saharan Africa | Yes |
| Guinea | Sub Saharan Africa | Yes |
| Mali | Sub Saharan Africa | Yes |
| Mauritania | Sub Saharan Africa | Yes |
| Mayotte | Sub Saharan Africa | Yes |
| Niger | Sub Saharan Africa | Yes |
| Senegal | Sub Saharan Africa | Yes |
| Sierra Leone | Sub Saharan Africa | Yes |
| Somalia | Sub Saharan Africa | Yes |
| Sudan | Sub Saharan Africa | Yes |
| Angola | Sub Saharan Africa | No |
| Benin | Sub Saharan Africa | No |
| Botswana | Sub Saharan Africa | No |
| Burundi | Sub Saharan Africa | No |
| Cameroon | Sub Saharan Africa | No |
| Cape Verde | Sub Saharan Africa | No |
| Central African Republic | Sub Saharan Africa | No |
| People's Republic of Congo | Sub Saharan Africa | No |
| Eritrea | Sub Saharan Africa | No |
| Ethiopia | Sub Saharan Africa | No |
| Gabon | Sub Saharan Africa | No |
| Ghana | Sub Saharan Africa | No |
| Guinea-Bissau | Sub Saharan Africa | No |
| Kenya | Sub Saharan Africa | No |
| Lesotho | Sub Saharan Africa | No |
| Liberia | Sub Saharan Africa | No |
| Madagascar | Sub Saharan Africa | No |
| Malawi | Sub Saharan Africa | No |
| Mauritius | Sub Saharan Africa | No |
| Mozambique | Sub Saharan Africa | No |
| Namibia | Sub Saharan Africa | No |
| Nigeria | Sub Saharan Africa | No |
| Reunion | Sub Saharan Africa | No |
| Rwanda | Sub Saharan Africa | No |
| Sao Tome and Principe | Sub Saharan Africa | No |
| Seychelles | Sub Saharan Africa | No |
| South Africa | Sub Saharan Africa | No |
| Swaziland | Sub Saharan Africa | No |
| Tanzania | Sub Saharan Africa | No |
| Togo | Sub Saharan Africa | No |
| Uganda | Sub Saharan Africa | No |
| Zambia | Sub Saharan Africa | No |
| Zimbabwe | Sub Saharan Africa | No |

| **Appendix 2: Description of Databases and variables derived** | |  |
| --- | --- | --- |
| **Database** | **Description** | **Variables** |
| Citizenship and Immigration Canada (CIC) | CIC includes demographic information about individuals’ at their entry into Ontario as permanent residents from 1985-2010. It excludes temporary residents (e.g. students, foreign workers and refugee claimants, those immigrants who landed after 2010, those who declared to move to another province but instead moved to Ontario, and those who could not be probabilistically linked too other databases. | Immigration status (economic, family, refugee or other)/ region of origin, length of stay. |
| Registered Persons Database (RPDB) | Includes residential and demographic information of all Ontario’s residents who are eligible for health care coverage. The eligibility includes being Canadian Citizens, landed immigrants or refugees; their primary and permanent residence is in Ontario; and physically reside in Ontario in any 12-month period for a minimum of least 153 days. For those born outside Ontario the health care coverage starts 3 months after their residency begins. | Demographic information for residents who are eligible for health care coverage. |
| Ontario Physicians’ Claims Database – OHIP Claims | Includes billing and diagnostic information submitted by approximately 95% of Ontario’s physicians. | Billings for cancer screening |
| ICES Physician Database (IPDB) | Comprises information from the Ontario  Health Insurance Plan (OHIP) about the health care providers including: demographics (training, year of graduation), specialization, and workload (type of work, place of work, location, payment plan, FTEs) | Physician demographics (age, sex), training, specialization, and workload (type of work, place of work, location, payment plan, FTEs) |
| Canadian Institute for Health Information Discharge Abstract Database (CIHI-DAD) | Includes acute in-patient hospital discharge data (i.e. demographic, administrative and clinical information) | Acute care hospital admissions  Comorbidities |
| The Client Agency Program Enrolment (CAPE) tables | This is a repository of the association of a registered person with a specific physician at a specific agency in a formally recognized program, including primary care Patient Enrolment Models. | Residents with primary care providers and type of primary care model |
| OHIP Corporate Provider Database **(**CPDB) | This is a provider registry which includes providers’ demographics and their organizations’ characteristics. It also includes providers’ credentials from the College of Physicians and Surgeons of Ontario (CPSO). | Provider’s organizations’ characteristics (PEM) |
| 2006 Canadian Census | The census provides [demographic](http://en.wikipedia.org/wiki/Demographics_of_Canada) and statistical data for all people living in Canada | Income neighbourhood |
